# Open up: a survey on open and non-anonymized peer reviewing

**DOI:** 10.1186/s41073-020-00094-z

**Published:** 2020-06-26

**Authors:** Lonni Besançon, Niklas Rönnberg, Jonas Löwgren, Jonathan P. Tennant, Matthew Cooper

**Affiliations:** 1 Linköping University, Norrköping, Sweden; 2 Université Paris Sud, Orsay, France; 3 Southern Denmark University Library, Campusvej 55, Odense, 5230 Denmark; 4 Center for Research and Interdisciplinarity, Universite de Paris, Rue Charles V, Paris, France; 5 Institute for Globally Distributed Open Research and Education, Ubud, Indonesia

**Keywords:** Peer review, Open science

## Abstract

**Background:**

Our aim is to highlight the benefits and limitations of open and non-anonymized peer review. Our argument is based on the literature and on responses to a survey on the reviewing process of alt.chi, a more or less open review track within the so-called Computer Human Interaction (CHI) conference, the predominant conference in the field of human-computer interaction. This track currently is the only implementation of an open peer review process in the field of human-computer interaction while, with the recent increase in interest in open scientific practices, open review is now being considered and used in other fields.

**Methods:**

We ran an online survey with 30 responses from alt.chi authors and reviewers, collecting quantitative data using multiple-choice questions and Likert scales. Qualitative data were collected using open questions.

**Results:**

Our main quantitative result is that respondents are more positive to open and non-anonymous reviewing for alt.chi than for other parts of the CHI conference. The qualitative data specifically highlight the benefits of open and transparent academic discussions. The data and scripts are available on https://osf.io/vuw7h/, and the figures and follow-up work on http://tiny.cc/OpenReviews.

**Conclusion:**

While the benefits are quite clear and the system is generally well-liked by alt.chi participants, they remain reluctant to see it used in other venues. This concurs with a number of recent studies that suggest a divergence between support for a more open review process and its practical implementation.

## Introduction

Pre-publication peer review of scientific articles is generally considered to be an essential part of ensuring the quality of scholarly research communications [1–3]. It can take many forms from single-round peer review, typical of conferences, to multiple-stage peer reviewing, more common in scholarly journals. Variants of these processes also include zero-blind (neither reviewers nor authors are anonymous), single-blind (reviewers are anonymous), and double-blind (both authors and reviewers are anonymous) systems (see for example [4]). With the major changes currently happening in scholarly communication systems, there is now a strong imperative for those who manage the peer review process to be absolutely clear about their policies and, where possible, upon what evidence such policies are based [5].

The names of these different variations can be confusing for researchers. While “open review” has often been used in the past to mean “non-anonymized” reviews (e.g., [6, 7]), we will use “open review” to refer to all reviews that are publicly available, whether anonymous or signed. Classical single/double-blind reviewing is held in high regard within scientific communities and is often considered as the gold standard for assessing the validity of research communications [1–3, 8–11]. Despite the criticism it sometimes incurs [12–18], peer review is still considered to be the “best that we have” [18] and only a few broad-scale attempts have been made to address the numerous issues with the current system, especially in human-computer interaction.

The alt.chi conference track, however, is an exception. It is a track within the annual Computer Human Interaction (CHI) conference, which is the predominant conference in the field of human-computer interaction. It started by offering papers rejected from the main track of CHI a second chance to be accepted through a set of different reviewers. The system then evolved into an open (publicly available) and non-anonymous process based on voluntary reviews. In 2013 and 2018, this approach was changed to a juried process where a small number of reviewers discussed the submissions, but in 2014 and 2019 reverted to the original open, volunteer-based and non-anonymous system.

In this article, our aim is to determine what advantages and limitations are presented by open peer reviewing through both a literature analysis and by gathering opinions from previous alt.chi authors as to what they value from such a system in comparison with the traditional single/double-blind review process. This offers a unique chance to explore an interesting system of peer review, to contribute to our developing understanding of this critical element of scholarly communication.

Even though this paper is based on a study of a specific conference track within a specific discipline, the outcomes of the study are easily transferable to other disciplines. The questions used in the survey are not specific in any way to the discipline or the conference, only to the nature of the review process and some of the alternatives which could be used.

## Related work

Of particular relevance to this discussion is past work on the topic of blind reviews, the benefits and challenges presented by open reviews, and the alternatives adopted in other research fields.

### Concerns with peer reviewing

While being almost as old as scholarship itself [19–21], peer review was only slowly formally introduced and established as the norm across the scholarly literature. In fact, one anecdote describes how Einstein chose to publish one of his papers in an alternative journal as an angry reaction to an anonymous peer review, and this may have been Einstein’s only actual encounter with peer review [19, 22]. While it is now well-established, peer review has often been criticized. Recent concerns include, but are not limited to (for more, see e.g., [18] or [23]), the lack of adequate training of reviewers, leading to them being unable to detect even major methodological errors [24]; the overall duration of the reviewing process which slows down progress in the scientific community [25, 26]; the unreliability of the assessments made by reviewers [27, 28]; the fact that interesting or important discussions and mitigation points highlighted by the review process are often not made accessible to other researchers [23]; that the review process is unable to prevent malicious, biased, or indifferent reviewers [14]; and that reviewers rarely receive appropriate credit for their reviews [23]. Noteworthy previous work has concluded that reviewers typically agree on a submitted manuscript at levels only slightly above chance [27] and that the current system of having two or three reviewers is unlikely to do much better than a lottery, based on mathematical modeling [29].

With respect to the CHI conference, Jansen et al. [30] conducted a survey of 46 CHI authors to determine what they value in the reviews they received in 2016. Jansen et al. noted that authors appreciated encouragement and having their work fairly assessed, but, at the same time, highlighted that authors sometimes found reviews to be unreasonable or insufficiently detailed. Jansen et al. also discussed and presented several points not covered by the reviewing guidelines (e.g., transparency about the statistical methods used or recommended and why) as well as several methods to make sure these guidelines for reviewers are followed during the reviewing process. The authors finally argued that non-public reviews make it hard to gather data to evaluate the peer review process and added that it could impede the development of Early Career Researchers (ERCs) who cannot find good examples of reviews from which to learn. These findings were echoed by Squazzoni et al. [31] who argued that the sharing of review data could both encourage and help reward reviewers.

### Types of peer review

Previous work has already investigated and attempted to summarize the main arguments for and against blinding, reciprocal or not, during peer review [6, 32, 33]. The four available and most commonly investigated options are zero-blind, single-blind, double-blind, and triple-blind. In a zero-blind system, authors, reviewers, and editors are aware of everyone’s identities (although the authors usually discover the identity of their reviewers only after the reviews are made available). In a single-blind system, only the identities of the reviewers are hidden from the authors, whereas double-blind systems also hide the identities of authors from the reviewers. In a triple-blind system, even the editor is blinded to the authors’ identities. It is sometimes believed that science benefits from increasing the level of anonymity.

Indeed, double-blind reviews have been shown by past research to be generally better than single-blind reviews [34–38]. It is thought to reduce reviewers’ biases [35, 36, 38] and to increase the number of accepted papers with female first authors in ecology or evolution journals [34] and seems to be preferred by both authors and reviewers [37]. Baccheli and Beller [39] showed that, despite the inherent costs of double-blind reviewing (e.g., difficulty for authors to blind papers and difficulty for reviewers to judge how incremental the work is), less than one third of the surveyed software engineering community disagreed with a switch from single-blind reviewing to double-blind reviewing. Prechelt et al. [16] investigated the perception of peer reviewing in the same community and reported that only one third of reviews are considered useful while the rest are seen as unhelpful or misleading. Many respondents to their survey supported the adoption of either double-blind or zero-blind reviewing.

With respect to the effectiveness of anonymizing authors, there is conflicting evidence [40]. Part of the literature argues that hiding their identity leads to better and less biased reviews [41–43], while it would seem that several large-scale studies do not support such claims [44–47]. Still, anonymizing authors appears to be one of the best solutions to address the known biases in research communities against female scientists and to increase the overall diversity of researchers engaged in the process [48–50].

Double-blind reviewing cannot, however, solve all the concerns previously mentioned, but open peer review might yield interesting solutions to some of these concerns.

### Towards (anonymous) open peer review

With all the recent publicity surrounding open research and open access publishing, it might seem that open peer reviewing is a relatively new idea. However, journals practising open reviews have existed since at least the 1990s [51] and the possible benefits of open peer reviews have been widely discussed in the literature (e.g., [52]). The sharing of review reports in one form or another actually even goes back to the origins of peer review itself [53]. The term “open review” is, however, loosely used and encompasses several elements [18, 54] that should be distinguished [55]: open identities, open reports, open participation, open interaction, open pre-review manuscripts, open final-version commenting, and use of open platforms. As stated in the introduction, in this manuscript, we wish to at least distinguish between openly available reviews and non-anonymized peer reviews. We feel that the best way for open peer review to progress is for different communities to advance the different elements outlined above, based on the best available evidence to them about what works best.

Jones [56] argued that anonymization could be detrimental because reviewers could act without fear of sanctions and suggested that reviews should be signed. This conclusion was later supported by Shapiro [57]. There are many variations on anonymity [23]. For example, the identities of reviewers could be revealed only on published papers while reviewers of rejected papers maintain their anonymity (as is the current practice in *Frontiers in Neuroscience* [58]), or reviewers could have to directly sign their reviews. Similarly, one has to distinguish between revealing the reviewers’ identities only to the authors or to the public by adding the names of the reviewers to the published manuscript, often (though not always) accompanied by their report and interactions with the authors. *PeerJ* gives the reviewers the option to add their names to their reports and the authors the possibility to add all interactions made during the reviewing process to the published manuscript [59] while *BMC Public Health* (and other BMC series) has made publication of signed reviews standard practice [60]. Yet another form of openness is to publish unsigned reviewers’ reports (which we define as open, anonymous peer review). This system is currently used by, for example, *The American Journal of Bioethics* [61].

The benefits of an open and/or non-anonymized reviewing system have been identified or postulated in previous work. Based on their investigation of peer review-based learning to foster learning of students with heterogeneous backgrounds, Pucker et al. [62] expected that “Reviewers might be more motivated thus producing better reports when they know that their reports will be published. In addition, errors in reviews could be identified and removed if a large number of peers are inspecting them.” Signed reviews have been evaluated as more polite and of higher quality when compared to anonymous reviews even though the duration of the reviewing process was found to be longer [52, 63].

## Method

Within human-computer interaction, we know of only one forum that uses an open review process: the alt.chi track within the CHI conference. Its initial purpose was to offer rejected papers from the primary submission process a second chance through another round of peer reviewing with new reviewers. Over the years, it has changed many times to include an open and public reviewing process or, in some years, a juried process. The procedure for open and public reviewing with open participation is the following:

- Authors submit a non-anonymized manuscript to a public forum.
- Anyone can submit a review or discuss the paper. Authors can invite reviewers.
- To ensure a sufficient number of reviews, authors of submissions are asked to review other submissions.
- Reviews are published non-anonymously. Anyone, including but not limited to authors and other reviewers, can see and respond to them until the system closes.
- The system is closed. The alt.chi conference committee decides which submissions to accept, and these accepted submissions are presented at the conference. In some cases, authors are asked to attach the reviews and discussions obtained during the process to the manuscript that will be published in the conference proceedings.

To better understand the advantages and limitations of such a review process in the human-computer interaction community, we asked previous authors to complete a short https://goo.gl/forms/ZPc1y4cin32NFZc43 on the reviewing system that was in place at alt.chi. We report the survey using the CHERRIES reporting guidelines [64]. The survey is an open survey targeted at previous alt.chi authors and reviewers or chairs.

### Administration

The survey, according to our institution’s rules, does not need to be approved by an IRB, but participants were informed about the purpose of the survey and its approximate completion time before they started answering it. The only personal information collected were the participants’ email addresses in order to inform them of the results of the study. They are stored in a separate file that only the authors can access. In addition to this, when the participants were done completing the survey, we gave them the opportunity to tell us if they did not want us to use their data and their answers (which occurred for just one participant whose answers were therefore discarded). The survey was presented as a Google form, and participation was voluntary. No incentives were directly offered, but we provided the opportunity to inform participants about the results of the study. The survey was distributed over five different pages. We did not implement a strategy to avoid multiple entries from a single individual, relying on researchers’ understanding of basic survey concepts and the importance of integrity when conducting such surveys.

### Recruitment

We first gathered the contact information of at least the first author of every accepted alt.chi paper from 2010 to 2018. We could not extract more information about the process (e.g., the number of submissions per year or the number of reviews they received) since the data are not available. When we believed that the first author of a publication could have already been the first author of an other publication, we also added the last author contact email to our list. We then sent an email to all identified contacts providing a link to the survey (in total 328 emails, 20 of which received direct Mail Delivery Errors, possibly because the authors changed their affiliations). Some of the authors we contacted have been involved in the organization of alt.chi before, and we know for sure that one of them replied to the survey (because data collection was anonymous, when respondents did not provide an email address, we cannot know whether or not they had been organizers/chairs). Additionally, we repeatedly posted a link on Twitter with the hashtag “chi2019” and asked people to forward the survey as much as possible. The online survey is still available, but closed to new responses. The Google form was accepting answers between December 3 and December 17, 2018, i.e., for a total duration of 14 days.

### Design and analysis

The survey comprised different categories of questions. The first category was about the person’s point of view as an author (Appendix 1). The second explored the person’s point of view as an alt.chi reviewer (Appendix 2). A final category (Appendix 3) evaluated how each respondent felt about the reviewing process and whether they would continue using it within alt.chi and even extend it to other tracks. In the last two questions, we also sought to gather additional comments about peer review and the questionnaire itself. All questions except the final two were mandatory. The analysis was not preregistered.

### Response rate and sample size

We gathered a total of 30 responses to our survey. We initially had 31 responses, but one respondent did not confirm that we could use their answers in a future publication so we removed their response from our data. If we do not consider the advertisement made on social media, our survey had a response rate of 9.7%.

While such a low number of respondents could be potentially seen as problematic, it appears through the literature that, in order to gather subjective measures and opinions, it can be enough. Indeed, Isenberg et al. [65] showed that, on average, between 1 and 5 participants are used in evaluation of research projects, while Caine [66] showed that among all CHI papers published in 1 year, all of the papers comprising user studies and therefore reporting on qualitative feedback and/or quantitative measures had less than 30 respondents/participants on average. Similar findings were reported in a more recent look at studies and participants [67]: in interviews or lab studies (both of which contain qualitative feedback and/or quantitative Likert-scale ratings), the majority of studies are conducted with fewer than 20 participants. In fact, for qualitative feedback and quantitative answers to Likert scales, the average is likely to be even lower and we found that often such research projects report results with 15 or less respondents (e.g., [68–74]), and sometimes with numbers as low as one (e.g., [70]) or two (e.g., [71]). Finally, we argue based on the literature that there is no meaningful cut-off point at which a sample size becomes inadequate or invalid because it would be “too small” [75] but instead the relationship between the value of a study and the size of the sample incrementally increases with each additional participant [75].

### Qualitative analysis

To limit interpretational biases when analyzing the answers to open-ended questions, one of the five present authors did a first pass to categorize each comment. Two other authors used these categories to classify the comments. We consider that an answer belongs to a category if two or more of the three authors classified it as belonging to that category. Our categorization spreadsheet is also available at https://osf.io/vuw7h/. Some participants gave responses which were more appropriate to the view of reviewers when asked about experiences as authors, and vice versa. Where this was apparent, the authors corrected this in the considerations of the data.

## Results

All anonymized answers (quantitative and qualitative) and scripts used on the data are available at https://osf.io/vuw7h/. Respondents had submitted an average of 1.9 papers (SD = 1.8) through the open reviewing process of alt.chi, while only two authors had submitted to a juried version of alt.chi. Most respondents (26 of 30, 86.7%) had submitted more than ten papers to more classical review tracks and were experienced with single/double-blind reviewing. The other four respondents had submitted between one and ten papers to other venues. Respondents had reviewed an average of 8.4 papers for alt.chi (SD = 10.1), while only three of them had reviewed for the juried process of alt.chi 2018. Most respondents (26 of 30, 86.7%) had reviewed more than ten papers in a single/double-blind review process while the remaining four had reviewed between one and ten papers within such a process. The final two questions obtained a response rate of 11/30 (which is reduced to 9/30 if we consider that two participants simply stated they had no additional comment) and 9/30 (similarly 8/30 with the statement of no additional comment).

### Qualitative feedback: limitations and advantages of the alt.chi reviewing process

Concerning the alt.chi process (before CHI2018) in particular, respondents highlighted that the reviewing could simply be a popularity contest, which in the end made individual reviews less relevant (7 of 30 respondents, 23.3%). One respondent replied that the “main limitation in my mind, is that when the reviewing is public the process might become a kind of popularity contest, or a test of who can bring the most supporters to the table.” Furthermore, in the alt.chi process, papers deemed uninteresting had less chance of acceptance as they would receive less reviews (4 of 30 respondents, 13.3%), and the limits of the invite-to-review (i.e., open participation) system were pointed out, as authors could invite friends to review (2 respondents, 6.7%).

Overall, respondents praised the discussions that the open review process of alt.chi (before CHI2018) brought, which is an advantage for both authors (13 of 30 respondents, 43.3%) and reviewers (14 of 30 respondents, 46.7%) and can also stimulate the discussions between reviewers (3 of 30 respondents, 10%). For example, one respondent stated that the open review process has the “[p]otential for discussion and critique between authors and reviewers during the review process, rather than the summative evaluation (accept / reject) in the full papers track.” The added transparency in the reviewing process was praised (5 of 30 respondents, 16.7%) as a benefit for authors as it helps them understand the comments from reviewers (2 of 30 respondents, 6.7%) and can reduce the *cite-me* effect (1 of 30 respondent, 3.3%). One respondent replied that “transparency is always welcome. I think reviewers are more constructive if their reviews are non-anonymous. Also the potential risk of reviewers asking ‘please quote me’ disappears.” The respondents mentioned that reviewers used a more polite tone (4 of 30 respondents, 13.3%), that the open review process fosters future collaborations as authors can directly contact reviewers and vice versa (2 of 30 respondents, 6.7%), and that the more diverse set of reviewers could also lead to interesting discussions (2 of 30 respondents, 6.7%). The respondents also highlighted that reviewers’ comments are usually better justified because reviewers are directly accountable for their reviews: this was seen as an advantage for both authors (6 of 30 respondents, 20%) and reviewers (8 of 30 respondents, 26.7%). As one respondent stated: “An actual discussion was possible [i.e. before CHI2018], and people mostly commented only if they actually had a well-founded opinion.” Interestingly, three respondents mentioned that signing reviews was a good way to receive credit for their work.

Considering open/public and non-anonymized reviewing, some respondents expressed concerns that reviewers might fear being truly critical and, consequently, self-censor their reviews (14 of 30 respondents, 46.7%) and that an author’s reputation could possibly directly influence the reviewer and the decision on the submission (4 of 30 respondents, 13.3%, as a limitation for authors, 2 of 30 respondents for reviewers, 6.7%). One respondent stated: “I think there is a lot of self-censorship and trying not to step on more senior people’s toes.” Finally, negative reviews, even if well-founded, could generate animosity and result in retaliation with respect to future submissions by the reviewer (4 of 30 respondents, 13.3%).

### Quantitative results: would the community consider this process for other CHI tracks?

**Fig. 1:**
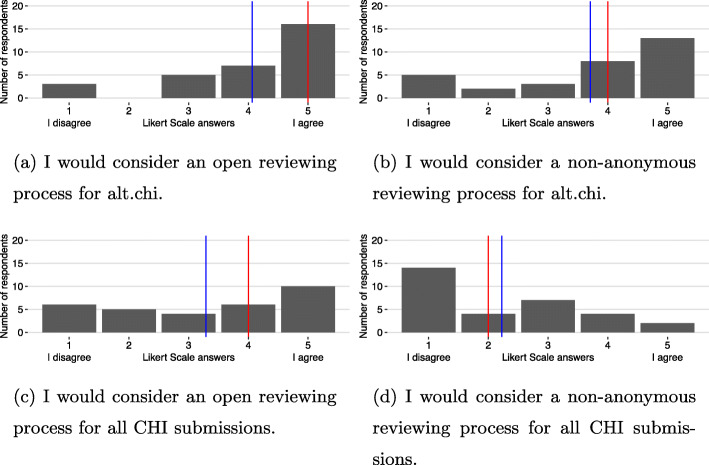
Results of the Likert-scale ratings for each question that participants were asked. The red bar indicates the median, the blue bar the mean

We have gathered the results of Likert-scale ratings (questions 11 to 14) in Fig. 1a to b. For all questions, a score of 1 indicates “I disagree” and a score of 5 “I agree.” We present these results with a bar chart showing the ranges of responses (as usually recommended [76]) in addition to means and medians. While the use of means for ordinal values has been initially slightly advocated against [77] and is still highly controversial [78], it appears in the literature that it is nonetheless highly used [79], useful to present [77, 78, 80, 81], and potentially even more useful than medians [80, 82].

The results in Fig. 1a and b highlight the openness and interest towards an open and non-anonymous review process that was already suggested by our qualitative results. Indeed, 23 respondents (of 30) gave a score of 4 or 5 (mean = 4.06, median = 5) to open review and 21 gave a score of 4 or 5 (mean= 3.71, median = 4) to non-anonymous reviews. This is not surprising since respondents have experience with this reviewing process for alt.chi. However, when asked whether they would consider such a process for the whole CHI conference, the results diverged from this. It seems that making reviews public (but not anonymous, Fig. 1c) could be envisioned, as 16 respondents would consider it and gave a score of 4 or 5 (mean = 3.29, median = 4). However, concerning the possibility to sign reviews, most respondents would not consider it: 18 gave scores of 1 or 2 (mean = 2.23, median = 2).

## Discussions

Our study’s qualitative and quantitative results suggest that the respondents have a general interest towards open and non-anonymous review processes. However, more than half of the respondents would nevertheless not consider signed reviews for other tracks of CHI. This might be due to the risk of retaliation for reviewers of a rejected paper, as mentioned by some of the respondents and echoing findings from previous work (e.g., [10, 55, 83]). Several possible procedures for non-anonymous reviews exist beyond simply asking reviewers to sign their reviews, however, such as giving the names of reviewers without attaching them to any specific report or only publishing the names of reviewers of accepted papers. Still, such alternatives are rarely used, and we hypothesize that they were probably not considered by most of our respondents (though future work could investigate this aspect further). Nonetheless, the reluctance to sign reviews for other CHI tracks contrasts with the rapidly growing number of journals that are using non-anonymous and public reviews (see, e.g., some of the BMC series [60] and the https://transpose-publishing.github.io site for a complete list).

The respondents indicated the limitations of the invite-to-review system, such as asking friends to review or turning the process into a popularity contest. Such problems are, however, not inherent to open and non-anonymous reviewing but rather emerge from the specific alt.chi implementation. An obvious improvement would to have a fixed number of assigned reviewers while still keeping the system open and non-anonymous.

The notion that reviewers might use a more polite tone when doing open reviews mirrors previous literature findings [52, 63], and it seems reasonable to assume that a more polite tone also could foster future collaborations between researchers. Some respondents pointed out that open reviews would make reviewer comments more justified as the reviewers would be directly accountable for their reviews (see also Jansen et al.’s [30] findings).

## Limitations and future work

While these results are interesting and could potentially help argue for opening the reviewing process to make reviews public, even if not signed, one has to take into account that respondents were all previously involved with alt.chi and should therefore be considered likely to be more open to the process than the rest of the community. It is therefore difficult to guarantee that the rather positive views towards open reviews would be shared by the larger CHI community. In addition, it should be noted that, even with our biased sample of previous alt.chi authors and reviewers, our results indicate that many of them consider that reviewers should remain anonymous in other CHI tracks or SIGCHI venues. This therefore suggests that the level of acceptance for broadening this practice even among researchers who have participated in open peer review before is quite low. We believe that this is a particularly interesting challenge that the open science community has to take into account: exposure to, and acceptance of, open systems or practices in specific contexts does not necessarily translate into other contexts.

A possible follow-up to our work could include gathering all the reviews and discussions generated through an instance of alt.chi and sharing it with the CHI community to produce a more diverse but informed opinion. In any case, future work includes polling authors and reviewers of the CHI community that do not participate in the alt.chi process in order to see if their opinions and ratings diverge from the ones of alt.chi participants. This could then be compared to peer review at conferences for other constituencies within the wider software engineering community.

## Conclusion

We have conducted an initial investigation on the perception of open reviewing within the only venue that has an open reviewing process in the human-computer interaction community. Our initial work highlighted that the non-anonymous open reviewing process adopted at alt.chi has some inherent flaws in its open participation design that could easily be addressed while maintaining the overall open and non-anonymous process. For instance, having a fixed number of assigned reviewers could solve many of the issues identified in the alt.chi system. From our results, it seems safe to assume that much of the alt.chi community values open and non-anonymous reviewing in general, but understanding the extent of this will require more work. It would also seem that the alt.chi community fears that the implementation of non-anonymous reviews in more prestigious venues could lead to issues such as biases towards accepting the work of more established researchers, self-censorship of reviews, or the possibility for authors to hold a grudge against their reviewers. While other scientific communities are starting to embrace the benefits of open and non-anonymous peer reviewing, the human-computer interaction community is using it only at alt.chi where accepted papers count only as extended abstracts rather than full archival publications in the proceedings of the conference. Indeed, our empirical findings seem to support the old adage that “double-blind peer review is the worst academic QA system, except for all the others.” It is nevertheless our hope that our work can contribute to further discussions on open peer reviewing processes and to experimentation with such processes in other academic venues. The small-scale survey implemented here could easily be adapted to help other scientific communities further understand and optimize their own peer review processes.

## Appendix 1

## Questions as an author

- How many papers have you submitted to alt.chi before CHI2018? (Open)
- How many papers have you submitted to alt.chi with the juried selection process (i.e., how many papers have you submitted to alt.chi in 2018)? (Open)
- How many papers have you already submitted to venues with a double/single blind reviewing process (i.e., for which reviewing was anonymous and not open)? (Possible answers: 0, 1–10, 10+)
- What do you think are the advantages for authors with the open/public and non-anonymized reviewing that was in place before CHI2018 when compared to the traditional double blind reviewing process? (Open)
- What do you think are the drawbacks/limitations for authors with the open/public and non-anonymized reviewing that was in place before CHI2018 when compared to the traditional double-blind reviewing process? (Open)

## Appendix 2

## Questions as a reviewer

- How many papers have you reviewed for alt.chi before CHI2018? (Open)
- Have you reviewed for alt.chi in 2018? (Yes or No)
- How many papers have you reviewed for other venues with a double/single blind reviewing process (i.e., for which reviewing was anonymous and not open)? (Possible answers: 0, 1–10, 10+)
- What do you think are the advantages for reviewers with the open/public and non-anonymized reviewing that was in place before CHI2018 when compared to the traditional double/single blind reviewing process? (Open)
- What do you think are the drawbacks/limitations for reviewers with the open/public and non-anonymized reviewing that was in place before CHI2018 when compared to the traditional double/single blind reviewing process? (Open)

## Appendix 3

## Additional questions

- I would consider an open/public (but possibly anonymous) reviewing process for all future alt.chi submissions. (Likert scale from 1 to 5 with 1 = "I disagree" and 5 = "I agree")
- I would consider a non-anonymized reviewing process for all future alt.chi submissions. (Likert scale from 1 to 5 with 1 = "I disagree" and 5 = "I agree")
- I would consider an open/public (but possibly anonymous) reviewing process for all CHI submissions. (Likert scale from 1 to 5 with 1 = "I disagree" and 5 = "I agree")
- I would consider a non-anonymized reviewing process for all CHI submissions. (Likert scale from 1 to 5 with 1 = "I disagree" and 5 = "I agree")
- If you wish to receive the results of our survey, you can enter your e-mail here. This information will not be used when making the data available. (Open Answer)
- Do you allow us to use the information you provided in future submission (once correctly anonymized)? (Possible answers: Yes or No)
- Do you have any additional comments on peer review ? (Open)
- Do you have any additional comments on the questionnaire itself? (Open)

## Data Availability

The data and scripts are available on https://osf.io/vuw7h/, and the figures and follow-up work on http://tiny.cc/OpenReviews.

## Abbreviations

*CHI*: The main venue for work done in human-computer interaction
